# Right Ventricle Remodeling and Function in Scleroderma Patients

**DOI:** 10.1155/2018/4528148

**Published:** 2018-03-20

**Authors:** Roxana Cucuruzac, Iolanda Muntean, Imre Benedek, Andras Mester, Nora Rat, Adriana Mitre, Monica Chitu, Theodora Benedek

**Affiliations:** University of Medicine and Pharmacy of Târgu Mureș, Gh. Marinescu Street, No. 38, 540139 Tîrgu Mureș, Romania

## Abstract

Scleroderma, known also as systemic sclerosis (SSc), is a severe disease associated with high mortality rates, and right ventricular (RV) remodeling and dysfunction, along with pulmonary artery hypertension (PAH), are among the most important internal organ manifestations of this disease. PAH has a higher prevalence in patients with SSc compared to the general population and represents a significant predictor of mortality in SSc. In patients with SSc, the morphological remodeling and alteration of RV function begin even before the setting of PAH and lead to development of a specific adaptive pattern of the RV which is different from the one recorded in patients with IAPH. These alterations cause worse outcomes and increased mortality rates in SSc patients. Early detection of RV dysfunction and remodeling is possible using modern imaging tools currently available and can indicate the initiation of specific therapeutic measures before installation of PAH. The aim of this review is to summarize the current knowledge related to mechanisms involved in the remodeling and functional alteration of the RV in SSc patients.

## 1. Introduction

Scleroderma, known also as systemic sclerosis (SSc), is a severe disease associated with high mortality rates, and right ventricular (RV) remodeling and dysfunction, along with pulmonary artery hypertension (PAH), are among the most important internal organ manifestations of this disease [1].

The mortality of SSc depends primary on the burden of the internal organ involvement. Pulmonary hypertension (PH), pulmonary fibrosis, and kidney damage are the most frequent causes of death in SSc [2, 3]. Myocardial, pericardial, and pulmonary artery (PA) involvement is observed in 1 out of 4 patients and serve as predictors of mortality in SSc, as approximately 30% of the SSc deaths are attributed to cardiac causes, followed by respiratory causes (17%) [4].

SSc is a complex autoimmune disease characterized by a marked chronic inflammation, which leads to increased connective tissue fibrosis and vascular involvement causing severe damage of internal organs (mainly the heart, lungs, gastrointestinal tract, kidneys, and muscles) and the skin [5].

Activation of the immune system, linked with fibroblast dysfunction, T lymphocyte, macrophage, and mast cell disturbances induce the alteration of the extracellular matrix by secretion of cytokines, chemokines, growth factors, and other potent mediators resulting in excessive collagen deposit in the tissues [6]. The exact etiology and trigger of the disease still remain unknown, but many studies suggest that genetic and environmental factors (silica, solvent, or radiation exposure) play an important role in the pathophysiology of SSc, generating changes in the expression of deoxyribonucleic acid (DNA) and microribonucleic acid (miRNA), which ultimately lead to inadequate activation of the immune system with phenotypic transformation of various cell types [7, 8]. Infiltration of the microvasculature accompanied by endothelial dysfunction and fibrosis initiates platelet activation and thrombosis causing ischemia of the surrounding tissues [9, 10]. It has been suggested that eNOS G894T polymorphism is correlated with an increased risk of SSc and PAH as well [11, 12].

The various clinical form of SSc varies from limited cutaneous systemic sclerosis (lcSSc) or CREST (calcinosis, Raynaud phenomenon, esophageal dysmotility, sclerodactyly, and telangiectasias) syndrome, to diffuse, sometimes fulminant forms with extended involvement of internal organs. The incidence of SSc greatly varies between different geographical regions and ethnic groups, with higher numbers among the African American population [13, 14]. The risk of developing SSc is significantly higher in woman than in men, with a 3–6 : 1 female to male ratio [15].

The aim of this review is to summarize the current knowledge related to mechanisms involved in the remodeling and functional alteration of the RV in SSc patients.

## 2. Systemic Sclerosis-Related Pulmonary Artery Hypertension (SSc-PAH)

PAH has a higher prevalence in patients with SSc compared to the general population, being more commonly observed in patients with lcSSc form, and represents a significant predictor of mortality in SSc [16, 17]. Current literature data suggests that the prevalence of PAH in this patient category reaches 12% and, despite the introduction of modern optimized treatments, the 3-year survival rate is still 52%, compared to 94% for SSc patients without PAH [18, 19]. At the same time one-year mortality rate of patients with SSc-PAH is around 30% versus 15% in patients with IPAH [20, 21]. Despite the fact that hemodynamic changes are less pronounced in SSc-PAH compared to idiopathic PAH (IPAH), SSc-related PAH is associated with twofold to threefold higher mortality risk, due to the systemic nature and complexity of the disease, added to a limited efficiency of the administered therapy [22, 23].

Argula et al. evaluated the functional changes of the RV during treatment in patients with SSc-PAH and IPAH at 3.8 and 1.95 years of follow-up. While the IPAH group showed significant improvement of the tricuspid annular plane systolic excursion (TAPSE [*p* = 0.01]), no such gain was observed in SSc-PAH patients, who also exhibited a worsening trend in tricuspid regurgitation jet velocity, right atrium (RA), and ventricular size [24].

In a comparative study Tedford et al. evidenced worse RV systolic function and prognosis in patients with SSc-PAH, compared to IPAH patients at similar RV afterload settings, suggesting the presence of an intrinsic systolic dysfunction in SSc patients [1]. The increase of the pulmonary artery pressure is a result of the alteration of the pulmonary microvascular bed triggered by the chronic inflammation, vasoconstriction, endothelial dysfunction, microthromboses, hypoxia, and excessive fibrous deposits at the level of the arterioles, causing increase of the pulmonary resistance [25, 26].

Hassoun et al. performed endomyocardial biopsies of the RV in SSc patients with and without PAH and compared these with samples from preserved ejection fraction heart failure (HFpEF) patients. Unpublished data from this study showed a significant decrease of the capillary density in SSc-PAH samples compared to the HFpEF and SSc without PAH samples. Furthermore, myocardial capillary density was significantly lower in samples from SSc patients with increased right atrial pressure (RAP), compared with lower RAP SSc samples, supporting the theory of structural RV alteration in these patients [27].

## 3. Right Ventricular Involvement in Scleroderma: A Distinctive Pattern

### 3.1. Right Ventricular Involvement in Scleroderma Patients without PAH

Cardiac manifestation of SSc includes involvement of the myocardium, pericardium, and the electrical conduction system, which may lead to ischemia, heart failure, pericardial effusion, and arrhythmias. RV failure can represent an important cause of mortality in SSc patients. It was believed that RV involvement is mainly linked to PAH, but recent studies suggest that SSc may have a direct impact on the RV structure and function, as alteration of RV function is more expressed in SSc-associated PAH than in non-SSc-PAH [28, 29]. The extent of cardiac involvement is likely to be underestimated, as autopsy studies identified considerable fibrotic changes of the myocardium in 70% of the examined patients [30]. Focal recurrent ischemia because of microvascular thrombosis, vasospasm thickening of the vascular wall, and fibrous deposits lead to irreversible functional and structural modifications of the myocardium [31, 32]. It is hard to differentiate the primary heart involvement from secondary development of these alterations due to PAH and kidney injuries. These changes may remain silent for long time (thus frequently underdiagnosed), but when clinically manifested it is described to represent an important negative prognostic factor [33].

Several studies tried to characterize RV involvement that occurs in SSc patients before development of PAH. Proper echocardiographic assessment of the RV function and volume estimation has been bound by its crescent shape, presence of intense trabeculations, and different contraction pattern, but novel techniques overcame these limitations [34]. Pigatto et al. evaluated the RV function of 45 SSc patients without any signs or symptoms of heart disease of PAH using three-dimensional echocardiography (3DE) and two-dimensional speckle-tracking echocardiography (2DSTE) and compared these findings with the similar parameters of 43 healthy subjects. A significant increase in RV size was observed in SSc patients with higher end-systolic volume [ESV, (*p* < 0.0001)], end-diastolic volume [EDV, (*p* = 0.049)], and reduced ejection fraction (*p* < 0.0001) determined by 3DE. Doppler measurements showed increased systolic pulmonary artery pressure (sPAP) and pulmonary vascular resistance (tPVR) in SSc patients compared to the control group. These changes were more pronounced in patients with lcSSc form [35].

Durmus et al. used 2DSTE for the assessment of RV function in patients with SSc without PAH. Significantly higher sPAP (within normal limits) was recorded in SSc patients (*p* = 0.002), accompanied by decreased RV systolic function with significantly lower TAPSE and tissue Doppler maximum systolic myocardial velocity (RVS′), but no correlation was observed between these parameters. Right ventricle global longitudinal strain (RVGLS) was also significantly lower in SSc patients compared to the control group (*p* < 0.001). An inverse correlation was observed between duration of the disease and TAPSE and RVGLS [36]. These findings consolidate the earlier results published by Schattke et al. who reported significantly decreased TAPSE and RVS′ determined by 2DSTE in SSc patients without PAH and identified isovolumetric acceleration (IVA) as the best predictor of early RV systolic impairment in this patient category [37].

Another early predictor of RV systolic dysfunction in SSc patients determined by 2DSTE may be represented by elevated longitudinal strain rates of the RV, which may serve as an adaptive response to even subtle elevation of the sPAP [38]. Furthermore these early systolic dysfunctions of the RV and RA may presage the sPAP elevation; thus it is important to detect them for the optimization of the further treatment of SSc patients, as recent trials show support for an early aggressive therapy [39–42].

### 3.2. The Distinctive Pattern of RV Remodeling and Function in Scleroderma Patients

**Figure 1:**
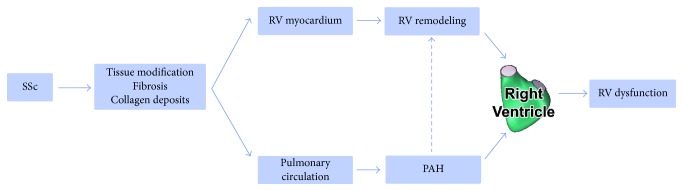
The complex interrelation between the mechanisms involved in development of RV dysfunction and remodeling.

Functional and structural alterations of the RV develop even before the setting of PAH in SSc patients, suggesting a remodeling pattern different from the one usually recorded in IPAH. Poor response to optimal therapy was observed in SSc-PAH, ultimately leading to RV failure and death, generating higher mortality rates compared to IPAH patients [1, 43, 44]. These findings led to the emergence of a novel theory about a different adaptive functional and structural remodeling of the RV in SSc patients. The complex interrelation between the mechanisms involved in development of RV dysfunction and remodeling is summarized in Figure 1. Different clinical studies tried to characterize the clinical condition of patients with SSc with or without PAH and to identify predictors of RV involvement and dysfunction. The main clinical studies and their findings are presented in Table 1.

In an autopsy study Overbeek et al. analyzed the histological samples of the RV from SSc-PAH and IPAH patients and compared them with samples taken from healthy controls. Significantly more inflammatory cells were observed in interstitium of the RV from SSc-PAH patients compared to IPAH patients and controls; however the quantity of fibrosis did not show significant differences between the groups, suggesting that the underlying mechanism of RV dysfunction may have multiple factors [45].

Kelemen et al. assessed the RV remodeling of SSc-PAH and IPAH patients using cardiac magnetic resonance imaging (cMRI). No significant differences were recorded in terms of RV mass, RV-EDV, RV ejection fraction, stroke volume, or TAPSE between the two groups [46]. It has been demonstrated that SSc-PAH patients exhibit significantly less pronounced increase of the RV mass (assessed by right ventricular modeling index (RVMI) and volume mass index (VMI)), in response to increased RV load (assessed by PVR and (mean) mPAP) compared to IPAH patients, evidencing a different adaptive hypertrophy mechanism of these patients [46]. However, the benefits and disadvantages gained from RV hypertrophy in this patient category are still under debate [47]. In a recently published article Ramjug et al. did not confirm the aforementioned findings, as no significant differences of RV mass were recorded between SSc-PAH and IPAH patients at increased RV load. Nevertheless, they found a notable correlation between the VMI and PVR in SSc-PAH through the entire range of PVR, which may be a predictor for survival, as earlier studies suggested [48, 49].

The functional reserve of RV may also play an important role in the adaptive remodeling of the RV in SSc. Hsu et al. assessed RV function and morphology during exercise (or atrial pacing) testing in 15 SSc-PAH and 9 IPAH patients with comparable resting RV parameters. The RV contractility of the IPAH group was significantly increased during exercise, while the SSc-PAH group did not display improved contractility leading to important increase of RV-EDV and RV-ESV. The authors attribute these changes to decreased calcium cycling in the myocytes of SSc-PAH group (*p* = 0.03). The abnormal RV-PA coupling and diminished force-frequency responsiveness (FFR) of these patients during exercise, along with reduced contractile and diastolic reserve, plead for the depletion of RV functional reserve of SSc-PAH patients. These results highlight the importance of intrinsic RV dysfunction, which ultimately leads to RV failure in SSc-PAH patients [50].

Kovacs et al. determined the pulmonary exercise hemodynamics of SSc patients with a 4-year follow-up period, observing significant increase of mPAP (*p* = 0.02) and PVR (*p* = 0.002) during exercise, with no changes in resting mPAP values. These findings illustrate the progressive nature of the disease and point out that alterations of exercise hemodynamic parameters precede the routinely determined resting values. The aforementioned changes might be related to the distinctive morphological and functional remodeling pattern of the RV in SSc patients [51].

## 4. Imaging Tools for Characterization of the RV in Scleroderma

Different imaging technologies are currently available for characterization of RF function and morphology, the most used ones being represented by echocardiography, scintigraphy, and cardiac magnetic resonance imaging (cMRI) (Table 1).

### 4.1. Echocardiography

Given the high prevalence of RV impairment in scleroderma patients, current guidelines recommend early echocardiographic screening of SSc patients at risk for PAH. Immediate initiation of optimal therapy and prevention of right heart failure can improve the prognosis and survival of patients with SSc-PAH [26]. Transthoracic echocardiography (TTE) is a widely available imaging method used for a proper functional and morphological assessment of the RV; however, a considerable number of patients remain undiagnosed until late stages of heart failure, presumably because of the complex geometry of the RV. Conventional 2D echocardiography along with Doppler technology is able to evaluate RV diameters, areas, and volumes, as well as pressures, velocities, and gradients across the cardiac chambers and valves [52].

Different echocardiographic biomarkers have been proposed for characterization of RV function and can serve for evaluation of ventricular dysfunction in patients with SSc. TAPSE is an easily determinable parameter which is considered the most accurate in the assessment of the global RV contractility (albeit it is influenced by load and structure). It correlates with the parameters obtained from right heart catheterization and was proved as a prognostic factor in SSc-PAH [53, 54]. Another useful parameter for the assessment of RV and global ventricular function is represented by the Tei index, or myocardial performance index (expressed as isovolumetric contraction time and isovolumetric relaxation time divided ejection time), which uses pulsed wave Doppler velocities of ventricular inflow and outflow to calculate ventricular performance. Several studies validated its correlation with lower survival rates in SSc patients [55–57]. Tissue Doppler imaging (TDI) has the advantage to determine myocardial velocities, allowing detection of systolic and diastolic deformations (defined as strain); therefore it is able to assess segmental and global RV function. The major drawback of this method is the dependence of the Doppler beam angle [34, 39].

Novel echocardiographic modalities based on speckle tracking technology allow a better identification of subclinical heart failure. The technique is a software-based method that allows the calculation of tissue velocity and deformations of the myocardium derived from 2D images, tracking pixels (speckles) of a certain myocardial sector along the cardiac cycle. It can detect the changes in length of the tracked segment during systole and diastole with calculation of strain and strain rate (SR) parameters. These parameters offer an accurate, operator, and angle-independent view of the segmental and global (systolic and diastolic) function of the ventricles. Multiple studies suggest that RV longitudinal strain values and patterns are helpful in the assessment of RV function in SSc and PAH patients and correlate well with the prognosis of this patient category [57–61].

### 4.2. Scintigraphy

The evaluation of the right ventricular function with nuclear imaging and scintigraphy can determine myocardial damage caused by SSc in early stages by identification of small perfusion defects. A ten-year survival study conducted by Steen et al. showed that SSc patients that presented perfusion defects upon scintigraphy examination with thallium presented a higher rate of cardiac disease and mortality [62]. Furthermore, 99 m Technetium ventriculography performed on 42 patients without PAH revealed a significantly lower RV ejection fraction compared to controls, both at baseline and at 2 hours from administration of 40 mg of oral Nicardipine [63]. Additionally, patients with SSc may present the so-called “myocardial Raynaud's phenomenon” that leads to transient ischemic episodes induced during the cold pressor test. Lekakis et al. performed dipyridamole-thallium-201 scintigraphy during cold pressor testing and found that scleroderma patients presented transient myocardial ischemia induced by cold and that subjects with Raynaud's phenomenon of under 5 years did not present any defects [64]. A combined echocardiography and scintigraphy research study by Papagoras et al. reported that 60% of patients presented reversible myocardial perfusion defects in spite of the lack of clinically active heart disease, even in younger patients [65].

Although scintigraphy has been proven to be a sensitive method for detection of early perfusion defects in systemic sclerosis patients with no cardiac symptomatology, further studies are needed to elucidate its role in establishing prognosis and therapeutic management, which may be a hard task, since nuclear imaging methods present high costs and are not widely available.

### 4.3. Cardiac MRI

cMRI is a noninvasive imaging modality that faced a tremendous development in the last decade, being able to provide high quality images on the anatomy and function of the heart [66–68]. cMRI has been shown to offer a significant aid in the diagnosis of several inflammatory processes affecting the myocardium as well as in fibrosis detection and quantification in a great number of pathologies, including SSc [69–73]. cMRI currently is the gold standard method for evaluation of RV parameters, and several studies have focused on the role of cardiac magnetic resonance for assessing right and left ventricular function, the extent and pattern of myocardial fibrosis, and perfusion abnormalities in SSc subjects [69, 70, 74]. Bezante et al. aimed to research the myocardial effects of SSc by cMRI imaging in 50 patients with scarce or no clinical signs of heart failure. The study showed that both the right and left ventricular ejection fractions were reduced compared to controls (*p* < 0.001 and *p* < 0.009, resp.), and the RV ejection fraction matched for body surface area was significantly reduced in subjects with diffuse compared to limited cutaneous SSc [74]. Tzelepis et al. sought to evaluate the distribution and pattern of the fibrotic involvement of the myocardium on 41 patients with SSc with the use of Delayed-Enhancement cMRI (DE-CMR) with gadolinium [70]. Their results revealed that 66% of the enrolled patients presented late enhancement, predominantly in the middle region of the myocardium wall, with a sparing of the endo- and epicardial regions, predominantly in the middle and basal areas of the left ventricle, with a noncoronary distribution. As for the right ventricular involvement, 17% of patients presented globular, intermittent enhanced areas in the RV insertion points, independent of the presence of PAH, upon echocardiographic assessment [70].

Hachulla et al. found that 75% of SSc patients presented cMRI abnormalities in their study population and that 21% of patients presented an altered RV ejection fraction, while 17% showed RV dilation in the absence of PAH. Moreover, they observed the thinning of the left ventricular myocardium that affected primarily patients with diffuse cutaneous SSc and those with no PAH, suggesting that this might be caused by disease's chronic effect on the microvasculature of the heart, being similar to the thinning that occurs during the ventricular remodeling process secondary to infarction [69].

Magnetic resonance imaging of the heart is also a useful tool in evaluating the effects of various therapies in patients with PAH, as well as for prognosis implications. Allanore et al. have evaluated the effect of bosentan on the perfusion and function of the myocardium by performing cMRI and Tissue Doppler Echocardiography on 18 SSc patients with no PA and no symptoms of impaired cardiac function. The study found that cMRI perfusion index and the echocardiography parameters were improved after 4 weeks of bosentan administration [75]. This shows that an improved myocardial function and perfusion can be revealed in SSc patients by highly sensitive imaging methods such as cMRI, which is a valuable noninvasive, nonirradiating, and highly reproducible imaging method useful in this patient population.

## 5. Conclusions

In patients with SSc, the morphological remodeling and alteration of RV function begin even before the setting of PAH and lead to development of a specific adaptive pattern of the RV which is different from the one recorded in patients with IAPH. These alterations cause worse outcomes and increased mortality rates in SSc patients. Early detection of RV dysfunction and remodeling is possible using modern imaging tools currently available and can indicate the initiation of specific therapeutic measures before installation of PAH.

## Figures and Tables

**Table 1:** Main clinical studies addressing Ssc in patients with or without PAH.

Imaging method	Author (year)	Number of patients	Clinical setting	Analyzed parameter	SSc	Controls	*p*
Echocardiography	Pigatto et al. (2015) [35]	*n* = 88	SSc versus healthy subjects	sPAP (mmHg)	33 ± 14.0	22 ± 5.0	<0.0001
				TAPSE (mm)	23 ± 3.0	26 ± 2.0	<0.0001
				PVR (WU)	1.9 ± 0.6	1.4 ± 0.3	0.001
				global RVLS (%)	−24.8 ± 4.0	−25.6 ± 3	n.s.
	Mukherjee et al. (2016) [58]	*n* = 178	SSc versus healthy subjects	sPAP (mmHg)	31.4 ± 13.3	22.6 ± 4.4	0.0001
				TAPSE (mm)	21.6 ± 4.7	22.5 ± 4.0	0.307
				PVR (WU)	1.48 ± 0.45	1.24 ± 0.26	0.002
				global RVLS (%)	−17.7 ± 5.9	−20.4 ± 2.4	0.005
	Durmus et al. (2015) [36]	*n* = 80	SSc versus healthy subjects	sPAP (mmHg)	24.2 ± 5.7	19.8 ± 6.2	0.002
				TAPSE (mm)	21.1 ± 3.2	24.3 ± 3.4	<0.001
				global RVLS (%)	−18.5 ± 4.9	−21.8 ± 2.4	<0.001

cMRI	Hachulla et al. (2009) [69]	*n* = 52	SSc-PAH versus SSc without PAH	RV hypertophy, *n* (%)	2 (17)	0 (0)	0.04
				RV dilation, *n* (%)	4 (33)	7 (17)	0.25
				Mean RV EF (%)	54 (13)	50 (11)	0.20
				Mean RV EDV index (ml/mm2)	75 (9)	79 (23)	0.67
				Delayed contrast enhancement, *n* (%)	1 (8)	10 (26)	0.42
	Tzelepis et al. (2007) [70]	*n* = 36	Abnormal versus normal 24-h ECG in SSc	Delayed contrast enhancement, *n* (%)	15 (78.9)	9 (52.9)	0.098
				Number of enhancing segments, *n*	5.4 ± 4.8	2.5 ± 2.9	0.035
				Enhancement at RV insertion points, *n* (%)	4 (21.1)	2 (11.8)	0.66
	Kelemen et al. (2015) [46]	*n* = 53	SSc-PAH versus IPAH	RV mass (g)	58.8	65.9	0.47
				RV EDV index (ml/mm2)	88.1	90.1	0.83
				RV EF (%)	46.0	41.6	0.29

Scintigraphy	Papagoras et al. (2014) [65]	*n* = 35	SSc patients	Reversible myocardial perfusion defects, *n* of pts (%)	21 (60)	-	-

sPAP: systolic pulmonary artery pressure; TAPSE: tricuspid annular plane systolic excursion; PVR: pulmonary vascular resistance; RVLS: right ventricle longitudinal strain; EF: ejection fraction; EDV: end diastolic volume.
